# Substituting effects of winged bean tuber-modified starches for cassava chip in concentrate diets on rumen fermentation, nutrient utilization, and blood metabolites in Thai native beef cattle

**DOI:** 10.5713/ab.23.0516

**Published:** 2024-05-07

**Authors:** Narirat Unnawong, Chaichana Suriyapha, Sompong Chankaew, Teppratan Rakvong, Anusorn Cherdthong

**Affiliations:** 1 Tropical Feed Resources Research and Development Center (TROFREC), Department of Animal Science, Faculty of Agriculture, Khon Kaen University, Khon Kaen 40002, Thailand; 2 Department of Agronomy, Faculty of Agriculture, Khon Kaen University, Khon Kaen 40002, Thailand

**Keywords:** Alternative Energy, Modified Starch, Physical Treatment, Ruminant, Winged Bean

## Abstract

**Objective:**

This study examined the effects of substituting winged bean tuber steam (WBTS) modified starches for cassava chips (CSC) in the concentrate diet on rumen fermentation, nutrient utilization, and blood metabolites in Thai-native beef cattle.

**Methods:**

Four Thai-native bulls were assigned randomly as a 4×4 Latin square design, which represents the amount of CSC replaced with WBTS in the concentrate mixture diets at 0%, 10%, 20%, and 30%.

**Results:**

Increasing levels of WBTS replacement for CSC in the concentrate diets had a quadratic effect on total dry matter (DM) intake (p<0.05). Replacement of WBTS at 20% and 30% for CSC did not alter total DM intake compared to 0% WBTS, whereas 10% WBTS replacement could significantly increase total DM intake by 0.41 kg DM/d compared to the control group. In addition, neutral detergent fiber (NDF) digestibility showed a quadratic increase (p<0.05) when CSC was substituted at various levels of WBTS in the concentrate diet (p<0.05). Replacement of CSC with WBTS at 10% and 20% showed higher NDF digestibility when compared to 0% replacement. There was a quadratic increase in blood glucose at 4 h post-feeding, and the average blood glucose value was significantly lower (p<0.01) when substituting CSC with WBTS. Substituting WBTS for CSC at 10% in the concentrate diet showed the highest blood glucose concentration when compared to other treatments. Replacing CSC with WBTS at 10% and 20% shows a higher concentration of C3 than those of other treatments (0% or 30%). The nitrogen (N) intake increased linearly (p<0.05) when substituting WBTS for CSC at all levels in the diet. Additionally, N retention and the ratio of N retention to N intake increased (p<0.05) when substituting WBTS for CSC at 10%, 20%, and 30% compared to 0%. The gross energy intake (GEI), digestible energy intake (DEI), and energy efficiency (DEI/GEI) were quadratically increased when substituted with various levels of WBTS for CSC in the concentrate diet.

**Conclusion:**

The findings indicate that substituting 10% of CSC in the concentrate diet with WBTS may be sufficient as an alternative feed resource for improving nutrient utilization and metabolic efficiency in beef cattle diets.

## INTRODUCTION

It is predicted that by 2050 there will be 10 billion people on earth. Producing enough food to satisfy demand is therefore crucial, particularly in the livestock system [1]. Therefore, it is essential to conduct research and seek alternative food sources to substitute primary animal feed ingredients to meet the rising demand for food and resolve the issue of seasonal feed shortages for animals. A widespread tuberous tropical field crop, cassava (*Manihot esculenta*), is farmed primarily in northeastern Thailand. For use as an energy source in feed, cassava roots can be cut, processed, and dried to create cassava chips (CSC). The starch content of over 90% of CSC is quickly and highly soluble in the rumen, with the CSC containing 80.16% non-fiber carbohydrate (NFC) as part of its dry matter (DM) [2]. In tropical regions, chewable feeds like CSCs are commonly utilized. However, the impacts of climate change on agricultural productivity have led to price instability across various feedstuffs, indicating a universal challenge beyond CSCs alone [3]. It is possible to increase local feed diversity and decrease feed restrictions in specific areas by including new tuberous species of plants or other plants [4].

Winged bean (*Psophocarpus tetragonolobus*; WB) has the potential to be used more extensively, especially in tropical regions. Unnawong et al [4] reported that WB tubers (WBT) consisted of crude protein (CP), ether extract (EE), neutral detergent fiber (NDF), and acid detergent fiber (ADF) at 19.01%, 1.23% 23.37%, and 5.93%, respectively. In addition, WBT contains high non-fiber carbohydrates (NFC) and gross energy (GE) at 53.10% and 3.87 kcal/g DM, respectively [4]. These high NFC and energy attributes make it a promising alternative for animal feed, particularly to replace CSC [4,5]. However, despite these advantages, it remains underutilized [6]. A recent study conducted by Suntara et al [3] revealed that substituting WBT for CSC at 100% resulted in a significant increase in gas production after 96 hours of *in vitro* ruminal fermentation, with no observed adverse effects. For high-producing ruminants to sustain high milk production and rapid weight increase, significant quantities of carbohydrate sources are typically fed. Nevertheless, ruminal pH drops, and the risk of rumen acidosis rises due to the quick fermentation of high-starch carbohydrates. As a result, a lot of studies have been conducted to determine the best means of modifying the rumen’s capacity to break down starch sources and increasing feed efficiency by shifting part of the digesting process to the small intestine [7].

Feed processing methods, such as physical and chemical methods, are thus used to modify starch to rumen degradation resistant starch [8–10]. Steaming is one of the physical methods for modifying starch into more economical and safer forms for animals, such as resistant starch, which leads to a decreased rate of starch breakdown in ruminant diets, ultimately enhancing starch digestion in the rumen [8]. Additionally, it was shown that modified CSC treated with steam had a higher capacity to alter the starch’s solubility and degradability in the rumen by reducing the starch’s degradation from CSC and raising its own degradability [7]. Increased growth performance in beef cattle was observed when the optimal modified starch level in the feed ranged from 15% to 25% of concentrate, while this level did not impact the rumen fermentation parameters [7,9]. A recent experiment conducted by Unnawong et al [4] showcased that employing steaming methods for starch modification in WBT could enhance ruminal *in vitro* pH and improve gas kinetics compared to untreated WBT. Furthermore, it was also discovered that steaming WBT did not influence *in vitro* degradability or the production of fermentation end-products when compared to steamed CSC. Maintaining ruminal pH and reducing or delaying ruminal starch degradability make the WBT modified by steam potentially a more successful feed efficiency technique [4].

However, previous research only tested the steamed WBT *in vitro* or *in sacco* studies. Thus, the evaluation of steamed WBT in *in vivo* studies is required to confirm and assess its practical use. It was hypothesized that a modified starch product derived from WBT may be utilized as a possible energy source to enhance starch digestion efficiency by maintaining the pH, ecology, and blood metabolites in the rumen, and balance starch fermentation in the rumen. The aim was to evaluate the effects of replacing CSC (no modified starches) with WBT-modified starches in diets on rumen fermentation, nutrient utilization, and blood metabolites in beef cattle.

## MATERIALS AND METHODS

### Ethical procedure

The study was conducted at Khon Kaen University’s Tropical Feed Resources Research and Development Center. The Ethical Committee approved all the research’s methods and procedures in accordance with Khon Kaen University’s institutional guidelines and those of the National Research Council of Thailand (record no. IACUC-KKU-46/65) to ensure animal welfare.

### Experimental station and treatment preparation

The experiment took place at the Tropical Feed Resources Research and Development Center (TROFREC), which is in Khon Kaen Province (16°26′48.16″N, 102°49′58.8″E) at the Department of Animal Sciences, Faculty of Agriculture, Khon Kaen University, Thailand. WBT was supplied by Khon Kaen University’s plant breeding researchers, while CSC was acquired from the university’s animal feed industry. The steam treatment process was altered, according to Srakaew et al [7]. Before being steamed in a two-tiered steam pot for 45 minutes at 100°C, the WBT were soaked in water for 12 hours to increase moisture content (20 cm high×80 cm^2^ in diameter) and left in the sun for 48 hours, or until the moisture content fell below 10%. Ultimately, all the CSC and WBTS were pulverized to the same particle size, and a 5 to 10 mm sieve was used to filter them out. All samples were then kept in storage for a later examination of their chemical makeup.

### Animals, treatments, and feeding

Four Thai native beef bulls, weighing 350±30 kg (body weight [BW]) were randomly placed in a 4×4 Latin square design (LSD) where the amount of CSC (no modified starches) replaced in the concentrate mixture diet by WBTS at 0%, 10%, 20%, and 30% of CSC, respectively. Under *ad libitum* feeding of rice straw (RS), each bull was provided the concentrate diet at 0.5% BW twice a day (8:00 am and 4:00 pm). Table 1 provides information on the chemical makeup of the concentrate diet, RS, CSC, and WBTS. Every animal was kept in a separate pen measuring 3×5 meters and equipped with cement water tanks. They were given a mineral block and unfettered access to clean water. Before the testing, the cattle were comparable in terms of age and weight. The cattle were given an injection of vitamin AD3E (Phenix; Anitech Total Solution Co., Ltd., Bangkok, Thailand), dewormed (Ivomec F; Kos Introtech Co., Ltd., Bangkok, Thailand), and weighed. The starting BW of the cattle was also recorded for each period. To ensure that the animals could easily adjust to the facilities, the experimental cattle were prepared in separate pens for a minimum of two weeks before the trial began. The study was conducted using healthy cattle. The study was split up into four 21-day periods. The first fourteen days were employed for treatment adaptation, and for the final seven days of each phase, the cattle were moved to the metabolism crates for total collection (feces and urine) to evaluate energy use, nitrogen (N) balance, and digestibility. Feed intake and refusals were calculated before morning feeding, and the results were recorded and computed daily. Body weights were recorded at the beginning and end of each period.

### Sample collection and analysis

Feed samples (both offered and refused) and feces were compiled during the last 7 days of each period for chemical composition analysis and to estimate of feed digestive capacities. The stool instances were sampled for around 5% of the total fresh weight and separated into two sections, the first for daily DM analysis and the second maintained in a refrigerator to determine nutritional content. By the end of the 7-day collection period, feed or fecal samples were thawed and combined for each animal. The combined samples were thoroughly mixed, and subsamples of offered feed, refused feed, and feces were dried in a forced-air oven at 60°C for 72 hours. After drying, the samples were ground using a Cyclotech mill (Tecator, Hoganas, Sweden) to achieve a particle size smaller than 1 mm. The urine was collected with a urine tube (Thermo Fisher Scientific Inc., Waltham, MA, USA) into 10-L containers with 10% H_2_SO_4_ and kept below 3.0 to prevent nitrogen loss. Every twenty-four hours, the urine tubes were changed, the amount of acidified urine was monitored, and an instantaneous 100-milliliter sample per animal was frozen at −20°C until analysis. All feed and fecal samples were analyzed to determine their DM (no. 967.03) and organic matter (OM, no. 492.05) content, following the procedure outlined in AOAC [11]. Feed, fecal, and urinary samples were analyzed to determine the quantity of N components using the Nitrogen Analyzer (Leco FP828 Nitrogen Analyzer; LECO Corporation, Saint Joseph, MI, USA) to assess the CP content. The NDF and ADF contents were measured by the ANKOM200 Fiber Analyzer following the procedure of Van Soest et al [12] (ANKOM Technology Corporation, Fairport, NY, USA). Using an auto-calculating bomb calorimeter (SHIMADZU CA-4PJ; SHIMADZU Corporation, Kyoto, Japan), the GE contents of the feed and fecal samples were calculated. The GE of feed intake and feces was used to compute the digestible energy intake (DEI). All procedures for the calculation of apparent digestibility, nitrogen balance, and energy utilization were done according to the report of Suriyapha et al [13] following:


Apparent digestibility (%)=(nutrient intake-nutrient in feces)×100/nutrient intake Apparent N absorption (g/d)=total N intake (g/d)-fecal N (g/d) Apparent N retention (g/d)=total N intake (g/d)-fecal N (g/d)+urine N(g/d) GEI (Mcal/d)=DM intake of diet (kg/d)×GE content in diet(Mcal/kg DM) DEI (Mcal/d)=GEI (Mcal/d)-FE (Mcal/d)

where GEI, gross energy intake; DEI, digestible energy intake; FE, fecal energy.

On the last day of each period, 45 mL of rumen fluid was collected at 0 and 4 hours after feeding, and the ruminal pH was immediately measured using a pH meter (Hanna Instruments HI 8424 microcomputer, Singapore). The equipment for rumen suction typically includes a specialized vacuum pump connected to a flexible tube designed for insertion into the rumen of an animal, along with various attachments such as collection containers and filters to facilitate the extraction and analysis of rumen contents. Two portions of the ruminal liquor were separated. The first portion (20 mL) was mixed with 5 mL of 1 M H_2_SO_4_ and stored at −20°C for ammonia nitrogen (NH_3_-N) measurement, utilizing a spectrophotometer (UV/VIS spectrophotometer, PG Instruments Ltd., London, UK) based on the Fawcett and Scott [14] method. Subsequently, volatile fatty acid (VFA) concentration analysis was conducted using a gas chromatograph (Newis GC-2030: Shimadzu, Shimadzu Corporation, Japan) equipped with a capillary column (DB-Wax column, 30 m length, 0.25 mm diameter, 0.25 m film; Agilent Technology, Santa Clara, CA, USA), following the instructions outlined by Porter and Murray [15]. In the second part, 1 mL of ruminal fluid was combined with 9 mL of formaldehyde solution, and the protozoal population was directly counted using a hemocytometer (Tiefe depth profundity 0.1 mm and 0.0025 mm^2^; ISO LAB Laborgerate GmbH, China) [16]. On the final day of each 10 mL of blood was extracted from the jugular vein (0 and 4 hours after feeding) and put into tubes containing 12 mg of ethylenediaminetetraacetic acid. Following the protocol of Crocker [17], they were then put on ice for 30 minutes before being centrifuged at 1,500 *g* for 15 minutes at 4°C to separate the plasma. They were then kept at −20°C until it was necessary to measure blood urea nitrogen (BUN). measuring blood glucose with commercial kits (Sigma Chemical Co., St. Louis, MI, USA; No. 640).

### Statistical analysis

The 4×4 LSD was analyzed statistically by employing SAS’s general linear model algorithm [18]. The following model was used to analyze the data:


$${\text{Y}}_{\text{ijk}}=\mu +{\text{M}}_{\text{i}}+{\text{A}}_{\text{j}}+{\text{P}}_{\text{k}}+{ɛ}_{\text{ijk}}$$

where: Y_ijk_, observation from animal j, receiving diet i, in period k; μ, the overall mean, M_i_, effect of the replacement levels (i = 0%, 10%, 20%, 30%), A_j_, the effect of animal (j = 1, 2, 3, 4), P_k_, the effect of period (k = 1, 2, 3, 4), and ɛ_ijk_ the residual effect. The standard error of the means is included with the mean values in the results. Duncan’s new multiple range test was used to compare treatment mean differences and means that differed by p<0.05 were considered statistically significant. For diet responses, orthogonal polynomials were found using both linear and quadratic effects.

## RESULTS

### Feeds, nutrient intake, and digestibility

Table 2 presents the impact of replacing CSC with WBTS in a concentrate mixture diet on feed intakes and nutrient intake, as well as the apparent digestibility of nutrients. Increasing levels of WBTS replacement for CSC in the concentrate diets had a quadratic effect on total DM intake (p<0.05), which ranged from 2.17% to 2.28% of BW. Replacement of WBTS at 20% and 30% for CSC did not alter total DM intake compared to 0% WBTS, whereas 10% WBTS replacement could significantly increase total DM intake by 0.41 kg DM/d compared to the control group. There was no change (p>0.05) in intake of RS, concentrate, and nutrient intakes except intake of CP, which was linearly increased when substituting various levels of WBTS (10%, 20%, and 30%) for CSC in the concentrate diet. In addition, NDF digestibility showed a quadratic increase (p<0.05) when CSC was substituted at various levels of WBTS in the concentrate diet (p<0.05). Replacement of CSC with WBTS at 10% and 20% showed higher NDF digestibility when compared to 0% replacement. However, there was no influence on the digestibility of DM, OM, CP, and ADF when replacing various levels of WBTS with CSC.

### Ruminal pH, ammonia nitrogen, protozoal population, and blood urea nitrogen

The ruminal pH, NH_3_-N concentration, protozoa populations, BUN, and blood glucose are shown in Table 3. There was no change in ruminal pH, NH_3_-N concentration, protozoal population, or BUN due to dietary treatment at 0 h and after 4 h of feeding time (p>0.05). However, there was a quadratic increase in blood glucose at 4 h post-feeding, and the average blood glucose value was significantly lower (p<0.01) when substituting CSC with WBTS in the concentrate diet. Substituting WBTS for CSC at 10% in the concentrate diet showed the highest blood glucose concentration when compared to other treatments.

### Volatile fatty acid profiles

The effect of WBTS replacing CSC in a concentrated diet on ruminal VFA is reported in Table 4. The total VFA at 4 hours post-feeding, as well as the average VFA values, showed a significant decrease (p<0.05) when WBTS was substituted for CSC at 20% and 30% in the concentrate mixture diet. Additionally, the C2 proportion at 4 hours post-feeding and the average C2 concentrations, as well as the ratio of C2:C3 at 4 hours post-feeding, exhibited quadratically decreased values when WBTS replaced CSC at 10% and 20% (p<0.05). Whereas the C3 concentration at 4 hours post-feeding and the average C3 concentrations exhibited a quadratic increase when WBTS was replaced with CSC in the diet at various levels (p<0.05). Replacing CSC with WBTS at 10% and 20% shows a higher concentration of C3 than those of other treatments (0% or 30%). However, there were no changes in the C4 proportion when replacing CSC with various levels of WBTS in the concentrate diet (p>0.05).

### Nitrogen utilization

The effect of the WBTS replacing CSC in the concentrate mixture on the nitrogen (N) balance is shown in Table 5. The N intake increased linearly (p<0.05) when substituting WBTS for CSC at all levels (10%, 20%, and 30%) in the diet. Additionally, N retention and the ratio of N retention to N intake increased (p<0.05) when substituting WBTS for CSC at 10%, 20%, and 30% compared to 0%. Furthermore, urine N excretion showed a quadratic decrease (p<0.05) when CSC was substituted with WBTS, with the lowest urine N excretion observed when 10% of WBTS replaced CSC in the concentrate diet (p<0.05). Nevertheless, there was no influence on fecal N excretion (p>0.05) when replacing various levels of WBTS for CSC.

### Energy utilization

Table 6 shows the effect of replacing CSC with WBTS on energy utilization in Thai-native beef cattle. The GEI, DEI, and energy efficiency (DEI/GEI) were quadratically increased when substituted with various levels of WBTS (10%, 20%, and 30%) for CSC in the concentrate diet, ranging from 0.9 to 1.39 Mcal/d, 11.36 to 12.41 Mcal/d, and 0.02 to 0.06, respectively. In contrast, the fecal energy (percentage of GEI) was linearly decreased when CSC was replaced by various levels of WBTS and was lowest by 14.82% when 10% WBTS replaced CSC (p<0.05) compared to the 0% WBTS group.

## DISCUSSION

### Feeds, nutrient intake, and digestibility

In this research, a greater total DM intake was observed when substituting 10% of the concentrate diet with WBTS compared to the control group (0% of WBTS in the diet). This increase might be attributed to the grain cereal being treated through many iterations of mechanical action, heat, and wetness to facilitate bacterial adhesion to starch granules. These processes cause the endosperm’s structure to collapse, the starch granules’ protein matrix to be disrupted, and the starch granules themselves to become gelatinized [4], which enhances the taste, odor, and fragrance of WBT, thereby improving palatability and feed intake [19]. Wang et al [20] reported that the steaming of sorghum in the diet expanded total intake, which may have been caused by the greater palatability of feed and enhanced ruminal fermentation by increased ruminal digestible starch. Furthermore, this processing gives starch a more accessible surface for both ruminal microbial and pancreatic enzymes, which is beneficial to microorganisms in their proliferation and may contribute to digestion in the rumen [21]. The result of this study revealed an increase in CP nutrient intake when replacing 10% to 30% of CSC with WBTS, compared to the unsubstituted group (0% of WBTS), possibly because of the higher CP content in WBTS compared to CSC [4]. The greater NDF digestibility observed in the substituting group (10% to 30% of WBTS) when compared to the control group could be attributed to the steam processing starch method used for WBTS. He et al [22] reported that the steaming process used in feed preparation could lead to a patchy, broken, irregular, and melted morphology of structural carbohydrates. This occurs due to the hydrolysis or melting of some cellulose and hemicellulose components on the surface when they are exposed to high temperatures [23]. Consequently, this process has the potential to alter and increase the specific surface area of the feedstuff, thereby enhancing feed utilization and digestibility [21,22,24]. Yahaghi et al [25] demonstrated that replacing barley with steam-treated sorghum grain in lamb diets could lead to improved ruminal fermentation and fiber digestibility. Similarly, Wang et al [20] found that substituting corn with steam-flaked sorghum in beef cattle diets resulted in enhanced NDF digestibility.

### Ruminal pH, ammonia-nitrogen protozoal population, and blood metabolites

The ruminal pH was not changed when CSC was replaced with WBTS in the diet. Generally, for maintaining normal rumen fermentation, average ruminal pH values should range from 6.2 to 7.0 [26]. In the present study, the ruminal pH was 6.86 to 6.93, which could indicate that high inclusion levels of WBTS (up to 30%) in diets are not detrimental to ruminal fermentation. It is probably due to WBTS affecting slow degradation and increasing the pH to over 6.0, which is less acidic and may positively affect ruminal pH [4,7,9]. Ramos et al [27] revealed that steaming treatments improved rumen fermentation by inducing a slow-fermented starch in the rumen, leading to a higher ruminal pH. Similarly, Unnawong et al [4] demonstrated that steam processing for starch modification could enhance and maintain ruminal pH in an *in vitro* trial.

Rumen degradable protein (RDP) and rumen undegradable protein are the two categories of proteins found in ruminants. Ruminal bacteria release enzymes that break down RDP, converting it into peptides, amino acids, and NH_3_-N [28]. The NH_3_-N is then converted into microbial CP (MCP) and flows through the liquid and solid phases of digesta to be absorbed in the intestine [4,7]. In this study, NH_3_-N did not change, which may be because CP digestibility was not influenced and ranged from 15.58 to 16.93 mg/dL by replacement with WBTS in the diet. According to McDonald and Ho [29], the range of ruminal NH_3_-N concentrations was 8.5 to 30 mg/dL. The ruminal NH_3_-N is the main source of nitrogen for the growth and reproduction of microbes in the rumen, as well as the main source of building blocks for protein production by bacteria [2,4]. According to Slyter et al [30], the minimal level of ruminal NH_3_-N needed for optimum ruminal microbial production in the rumen is typically 5 mg/dL. This research suggests that the rumen microbiome has enough NH_3_-N to support the synthesis of microbial proteins.

In this study, the BUN levels ranged from 10.50 to 10.75 mg/dL, falling within the normal range for tropical ruminants, which typically varies from 6.30 to 25.50 mg/dL [13]. The concentration of ruminal NH_3_-N, as well as nutrition, age, and protein consumption, all have an impact on this range [13]. Our result reveals no significant impact on BUN concentration in diets substituting corn with WBTS. This might be the case because the substitution of WBTS in the diet had no effect on NH_3_-N and because BUN concentration is associated with ruminal NH_3_-N generation [3,10].

In this study, blood glucose concentrations ranged from 68.65 to 77.71 mg/dL, which is close to the normal range. Srakaew et al [7] reported that glucose levels in the bloodstream were within the normal range of 45 to 75 mg/dL. Our study demonstrated that blood glucose levels increased at 4 hours post-feeding in beef cattle when CSC was substituted for WBTS at 10% in the diet. These increases might be influenced by a greater propionic acid (C3) proportion. According to Qiao et al [31] and Zhong et al [32], steaming techniques can also be used to control the rate of starch degradation. This increases the amount of starch available in the small intestine and rumen, which can improve the C3 proportion and raise levels of blood glucose. In the study conducted by Rastgoo et al [33], it was shown that the use of steam corn as a substitute for ground corn in the corn grain processing technique resulted in elevated levels of blood glucose and C3 concentration. Similarly, Srakaew et al [7] reported that steam-flaked corn provided greater availability of starch and glucogenic products (C3 production) when compared with fine-ground corn. However, replacing 20% to 30% of CSC with WBTS in the diet restored blood glucose levels to the normal range (45 to 75 mg/dL). Normally, the glucose blood level is maintained in a normal range. When glucose levels deviate from the specified range, the pancreas secretes insulin and glucagon to regulate and restore glucose levels to within the normal range. Additionally, glucose is stored in the form of glycogen in skeletal muscle and liver cells [6].

### Volatile fatty acid profiles

The present study shows that the complete substitution of CSC with WBTS results in 10% to 20% increased concentrations of ruminal C3 proportion, whereas decreased proportions of C2 are due to the fact that in the rumen the metabolic pathways for C3 creation are more effective at transforming energy than those for C2 and C4 production [3,10]. He et al [22] suggested that ruminants may benefit from a more effective source of energy supply if they were fed steam feedstuffs. It’s probable that the steam-flaking process broke down the protein’s disulfide bond and the starch’s embedded structure, making the starch surface more exposed to ruminal microbial breakdown enzymes [20,21], hence enhancing the starch’s ruminal degradability when fed at a replacement optimal level of 10% to 25% of concentrate [7,21]. According to Qiao et al [31], steam-flaking cereal grains cause the concentration of C3 to rise while the ratio of C2 to C3 decreases. According to Makizadeh et al [34], slow-fermented starch treated with steam produced high short-chain fatty acids. Similarly, He et al [22] suggested that steam processing of millet stalks and sugarcane tips could decrease the C2:C3 molar ratio and increase the molar proportion of C3. However, flaking and steaming changed the amylopectin in grains or cereals by turning it into gelatin and breaking it up into branches. This increased the amount of amylose in the starch, which made the starch granules stickier and thicker, a property called resistant starch. When a diet includes a high replacement level of resistant starch (over 30%), the starch’s solubility decreases, and it breaks down less in the rumen [19]. This slower breakdown in the rumen could explain why the total VFA and C3 proportions were lower when WBTS was 20% and 30% of the diet, respectively, in this study. Wang et al [20] revealed that the incorporation of steam-flaked sorghum in lamb diets apparently decreased ruminal VFA concentrations when replacing a high steam-flaked (>30%) sorghum diet, and the ratio of C2 to C3 remained influenced. Similarly, Zhong et al [32] and Rafiee-Yarandi et al [35] showed a slight decrease in total VFA and proportion of ruminal C3. It appears that less starch may have been digested in the rumen of cows fed steam corn (20% to 40%) than those fed ground corn.

### Nitrogen utilization

In this investigation, the results indicated that steam processing could increase nitrogen (N) intake (g/d). The N intake in this study varied from 1.10 to 1.17 g of N/kg BW^0.75^/d, which was higher than the optimal recommended value (0.68 to 0.86 g of N/kg BW^0.75^/d) and provided sufficient protein intake for maintenance in Thai-native male cattle fed RS-based diets [6]. This increase in CP intake was likely due to the correlation between concentrates and the higher N content when substituting WBTS for CSC. In this study, replacing CSC with WBTS indicated a lower urinary N excretion, particularly at the 10% level, which significantly reduced urinary N excretion up to 23.54% compared to the group without replacement. It could be because substituting WBTS for CSC does not affect ruminal NH_3_-N concentrations [31]. Additionally, the lower urinary N excretion in the steam processing diets might indicate a higher N utilization when cattle are fed the steam diets compared with the raw material diets [9,19,20]. Furthermore, this could be attributed to the increase in ruminal C3 levels, leading to a subsequent reduction in urinary nitrogen losses by decreasing the utilization of amino acids for gluconeogenesis [19,20]. Lemosquet et al [36] reported that the use of some amino acids for the process of gluconeogenesis increases when the dietary supply of starch is low. The increase in C3 levels suggests a greater supply of glucose precursors might inhibit hepatic gluconeogenesis by amino acid deamination [37]. According to a study conducted by Miyaji et al [37], the inclusion of higher amounts of steam brown rice in the diet was shown to have a positive impact on N retention, and the presence of glucogenic substances such as C3 and plasma glucose concentration as well as this dietary modification was seen to decrease urinary N losses by mitigating the process of deamination. Similarly, previous studies [33,34] demonstrated that steamed corn decreased urinary N excretion in steers. However, there were no changes in the fecal N excretions by dietary treatment, so replacing CSC with WBTS (0% to 30%) probably did not affect the nutrient digestibility of CP. In this investigation, the average values of nitrogen retention and absorption were 48.07 g/d (53.93 to 62.35) and 41.98 g/d (45.36 to 56.14), respectively, which were close to the range or utilizable for Thai-native cattle fed RS-based diets [13]. When substituting WBTS for CSC at 10% to 30% in the concentrate diet, it indicated greater N absorption and N retention. This could be explained by a greater N intake and lower N excretion. The levels of N intake and defecation are correlated with concentrates and higher CP levels, as well as their impact on N absorption and retention [13].

### Energy utilization

This study’s results indicated no adverse effect on energy utilization when substituting WBTS for CSC in Thai-native beef cattle. Previous studies [7,13] reported that Thai-native cattle weighing 350 kg required a daily GEI ranging from 14.20 to 16.20 Mcal/d to ensure that the animals gained adequate energy to support both their body maintenance and growth. In the present study, GEI increased when CSC was substituted with WBTS, possibly attributed to the greater total intake enhancing GEI. The study found that the fecal energy excretion (% of GEI) in Thai-native cattle went down when replacing CSC with WBTS in the diet. Meanwhile, there was greater digestible energy (DE) in Mcal/d when CSC was replaced with WBTS at 10% in the diet. It is probably because a favorable correlation was observed between increases in DE intake and decreases in energy loss from feces. This is because the energy content rose as the amount of WBTS in the diet increased, and the replacement of WBTS by CSC led to significant changes in the NDF digestibility. The steam treatment of grain increased the amount of soluble non-starch polysaccharides and the degree to which starch gelatinized [31,32]. This process could change and increase the feedstuff’s specific surface area, which would make it easier for animals to use and digest [7,9,33]. Plascencia et al [38], demonstrated that steam treatment resulting in puffing had positive effects on the nutrient digestibility of grain and reduced the amount of energy wasted by ruminants’ excrement. Moreover, a significant factor in enhancing ruminal fermentation, microbial protein synthesis, and nutrient digestibility may potentially be the synchronization of the rate of dietary energy degradation and protein release in the rumen and their outcomes [19,34,35]. In this study, the results showed a positive effect on the digestibility of NDF. Blood glucose, N absorption, N retention, and C3 proportion were increased. Thus, WBTS is a better energy source because, in comparison to CSC, it has a higher protein and carbohydrate content.

## CONCLUSION

In conclusion, the replacement of WBTS for CSC in concentrate diets exhibited notable improvements in total DM intake, CP intake, and NDF digestibility. Blood glucose levels, ruminal C3 concentrations, nitrogen utilization, and energy efficiency could be enhanced when WBTS replaced CSC. The results suggest that replacing 10% of CSC with WBTS in the concentrate diet might suffice as an alternative feed resource, enhancing nutrient utilization and metabolic efficiency in beef cattle diets. However, further studies on the effects of replacing CSC with WBTS in the diet of high-producing dairy cows are necessary to confirm the advantages of utilizing alternative livestock feed.

## Figures and Tables

**Table 1:** Ingredients and chemical composition of concentrate, rice straw, cassava chips and winged bean tuber

Items	Level of WBTS replacing CSC (% DM)				RS	CSC	WBTS

	0	10	20	30			
Ingredient (% DM)							
Cassava chip	40	36	32	28	-	-	-
Soybean meal	20	20	20	20	-	-	-
Rice bran	18	18	18	18	-	-	-
Palm kernel meal	18	18	18	18	-	-	-
WBTS	0	4	8	12	-	-	-
Urea	1.0	1.0	1.0.	1.0	-	-	-
Minerals and vitamins^1)^	1.0	1.0	1.0	1.0	-	-	-
Molasses	1.0	1.0	1.0	1.0	-	-	-
Salt	1.0	1.0	1.0	1.0	-	-	-
Chemical composition							
DM (%)	91.44	91.47	91.82	92.45	93.78	91.78	92.50
Organic matter (% DM)	95.81	95.86	95.42	94.53	88.80	94.68	97.00
Crude protein (% DM)	16.85	17.30	17.77	18.20	4.40	2.90	22.50
Neutral detergent fiber (% DM)	23.42	24.56	24.70	25.59	73.34	36.78	31.37
Acid detergent fiber (% DM)	10.50	11.27	11.50	12.06	44.99	18.47	14.43
GE (kcal/g DM)	3.84	3.83	3.87	3.80	3.40	3.88	3.85

WBTS, winged bean tuber steam; CSC, cassava chips; DM, dry matter; RS, rice straw; GE, gross energy.

1) Minerals and vitamins (each kg contains): vitamin A, 10,000,000 IU; vitamin E, 70,000 IU; vitamin D, 1,600,000 IU; Fe, 50 g; Zn, 40 g; Mn, 40 g; Co, 0.1 g; Cu, 10 g; Se, 0.1 g; I, 0.5 g.

**Table 2:** Substituting effect of the winged bean tuber modified-starches for cassava chip in concentrate mixture on feed intake and rumen fermentation in Thai native beef cattle

Items	Level of WBTS replacing CSC (% DM)				SEM	Contrast	
	
	0	10	20	30		L	Q
Rice straw intake							
kg DM/d	5.71	6.10	6.01	5.95	0.153	0.20	0.06
% of BW	1.69	1.77	1.71	1.67	0.052	0.49	0.08
g/kg BW^0.75^	71.32	75.56	74.10	73.70	1.853	0.35	0.10
Concentrate intake							
kg DM/d	1.76	1.78	1.81	1.80	0.064	0.09	0.55
% of BW	0.52	0.51	0.52	0.50	0.112	0.42	0.72
g/kg BW^0.75^	22.60	22.04	22.20	21.83	1.482	0.18	0.78
Total intake							
kg DM/d	7.47^b^	7.88^a^	7.82^ab^	7.75^ab^	0.987	0.13	0.04
% of BW	2.21^b^	2.28^a^	2.23^ab^	2.17^ab^	0.005	0.88	0.04
g/kg BW^0.75^	93.35	98.40	97.29	95.77	2.191	0.39	0.05
Nutrient intake (kg DM/d)							
Organic matter	6.76	7.12	7.06	6.99	0.218	0.11	0.47
Crude protein	0.55^b^	0.58^a^	0.59^a^	0.59^a^	0.009	<0.01	0.13
Neutral detergent fiber	4.60	4.91	4.85	4.82	0.112	1.00	0.06
Acid detergent fiber	2.75	2.94	2.91	2.89	1.481	0.47	0.53
Nutrient digestibility							
DM (%)	62.05	64.61	64.56	66.93	2.361	0.07	0.95
Organic matter (% DM)	64.86	67.01	67.39	69.09	2.203	0.08	0.88
Crude protein (% DM)	66.29	66.26	67.65	68.11	2.162	0.33	0.87
Neutral detergent fiber (% DM)	56.62^b^	63.80^a^	62.19^a^	61.13^ab^	2.380	0.14	0.03
Acid detergent fiber (% DM)	54.42	55.92	54.86	59.73	3.634	0.22	0.52

WBTS, winged bean tuber steam; CSC, cassava chips; SEM, standard error of the mean; L, linear; Q, quadratic; DM, dry matter; BW, body weight.

a,b Means within a row with different superscripts differ significantly (p<0.05).

**Table 3:** Substituting effect of the winged bean tuber modified-starches for cassava chip in concentrate mixture on ruminal pH, NH_3_-N concentration, and ruminal protozoal population in Thai-native beef cattle

Items	Level of WBTS replacing CSC (% DM)				SEM	Contrast	
	
	0	10	20	30		L	Q
Ruminal pH							
0 h post feeding	7.18	7.18	7.18	7.20	0.022	0.68	0.67
4 h post feeding	6.87	6.93	6.92	6.86	0.043	0.10	0.70
Average	7.03	7.06	7.05	7.03	0.031	0.25	0.80
Ruminal NH_3_-N (mg/dL)							
0 h post feeding	13.85	14.34	15.05	14.93	0.590	0.06	0.48
4 h post feeding	17.31	18.78	18.81	18.34	0.742	0.21	0.09
Average	15.58	16.56	16.93	16.64	0.562	0.07	0.13
Protozoal count (log_10_ cell/mL)							
0 h post feeding	5.89	5.87	5.90	5.94	0.041	0.06	0.17
4 h post feeding	6.02	6.04	6.04	6.06	0.023	0.06	0.10
Average	5.96	5.96	5.97	6.00	0.032	0.15	0.76
Blood urea-N (mg/dL)							
0 h post feeding	8.25	8.00	8.38	8.25	0.392	0.76	0.82
4 h post feeding	12.75	12.81	13.13	13.25	2.281	0.06	0.87
Average	10.50	10.41	10.75	10.75	0.202	0.10	0.74
Blood glucose ( mg/dL)							
0 h post feeding	68.65	71.42	70.16	69.14	0.950	0.41	0.08
4 h post feeding	74.38^b^	77.71^a^	75.54^b^	74.32^b^	0.621	0.25	<0.01
Average	71.52^c^	74.57^a^	72.85^b^	71.73^c^	0.831	0.11	<0.01

WBTS, winged bean tuber steam; CSC, cassava chip; SEM, standard error of the mean; L, linear; Q, quadratic.

a–c Means within a row with different superscripts differ significantly (p<0.05).

**Table 4:** Substituting effect of the winged bean tuber modified-starches for cassava chip in concentrate mixture on volatile fatty acids profiles in Thai-native beef cattle

Items	Level of WBTS replacing CSC (% DM)				SEM	Contrast	
	
	0	10	20	30		L	Q
Total volatile fatty acid (mM)							
0 h post feeding	83.58	85.53	83.92	84.37	0.851	0.78	0.23
4 h post feeding	114.03^a^	117.28^a^	94.26^b^	92.24^b^	5.132	0.78	<0.01
Average	98.98^a^	101.41^a^	87.92^b^	89.30^b^	2.872	0.85	<0.01
Acetic acid (mol/100 mol)							
0 h post feeding	68.61	68.06	67.91	68.14	0.341	0.17	0.13
4 h post feeding	70.05^a^	67.21^c^	68.35^bc^	69.24^ab^	0.643	0.53	<0.01
Average	69.33^a^	67.64^c^	68.13^bc^	68.69^ab^	0.332	0.20	<0.01
Propionic acid (mol/100 mol)							
0 h post feeding	18.92	19.93	19.47	19.40	0.492	0.54	0.14
4 h post feeding	21.16^b^	24.27^a^	23.81^a^	21.48^b^	0.801	0.85	<0.01
Average	20.04^b^	22.10^a^	21.64^a^	20.44^b^	0.490	0.64	<0.01
Butyric acid (mol/100 mol)							
0 h post feeding	12.47	12.01	12.62	12.46	0.471	0.70	0.66
4 h post feeding	8.79	8.52	7.84	9.28	0.603	0.68	0.06
Average	10.63	10.26	10.23	10.87	0.342	0.18	0.38
Acetic acid:propionic acid ratio							
0 h post feeding	3.63	3.41	3.49	3.51	0.102	0.42	0.11
4 h post feeding	3.31^a^	2.77^b^	2.87^b^	3.25^a^	0.141	0.82	<0.01
Average	3.47	3.09	3.18	3.37	0.112	0.12	0.09

WBTS, winged bean tuber steam; CSC, cassava chip; SEM, standard error of the mean; L, linear; Q, quadratic.

a–c Means within a row with different superscripts differ significantly (p<0.05).

**Table 5:** Substituting effect of the winged bean tuber modified-starches for cassava chip in concentrate mixture on nitrogen (N) balance in Thai-native beef cattle

Items	Level of WBTS replacement CSC (% DM)				SEM	Contrast	
	
	0	10	20	30		L	Q
NI							
g/d	87.64^b^	92.21^a^	93.77^a^	93.30^a^	1.221	0.04	0.09
g/kg BW^0.75^	1.10^b^	1.14^ab^	1.16^a^	1.17^a^	0.022	<0.01	0.29
NE (g/d)							
Fecal NE	33.71	29.86	31.99	32.88	1.673	0.95	0.06
Urinary NE	8.57^a^	6.21^c^	7.04^b^	7.67^b^	0.261	0.65	0.02
N absorption							
g/d	53.93^b^	62.35^a^	61.78^a^	60.42^ab^	2.232	0.10	0.02
g/kg BW^0.75^	0.68^b^	0.77^a^	0.76^a^	0.76^a^	0.032	0.14	0.02
NR							
g/d	45.36^b^	56.14^a^	54.74^a^	52.75^a^	1.624	0.15	0.02
g/kg BW^0.75^	0.68	0.69	0.68	0.67	0.182	0.43	0.33
NR/NI	0.52^b^	0.61^a^	0.58^a^	0.57^a^	0.023	0.05	<0.01

WBTS, winged bean tuber; CSC, cassava chip; SEM, standard error of the mean; L, linear; Q, quadratic; NI, nitrogen intake; BW, body weight; NE, N excretion; NR, N retention.

a–c Means within a row with different superscripts differ significantly (p<0.05).

**Table 6:** Substituting effect of the winged bean tuber modified-starches for cassava chip in concentrate mixture on energy utilization in Thai-native beef cattle

Items	Level of WBTS replacing CSC (% DM)				SEM	Contrast	
	
	0	10	20	30		L	Q
GEI							
Mcal/d	26.17^b^	27.56^a^	27.44^a^	27.07^a^	0.431	0.03	0.04
Mcal/kg BW^0.75^	0.33	0.34	0.34	0.34	0.007	0.13	0.25
Faecal energy							
Mcal/d	9.89	8.87	9.80	9.78	0.502	0.72	0.19
% Gross energy intake	37.79^a^	32.19^b^	35.72^ab^	36.13^ab^	1.072	0.04	0.73
Mcal/kg BW^0.75^	0.12	0.11	0.12	0.12	0.005	0.38	0.33
DEI							
Mcal/d	16.28^b^	18.69^a^	17.64^ab^	17.29^ab^	0.581	0.69	0.04
Mcal/kg BW^0.75^	0.21	0.23	0.22	0.22	0.014	0.19	0.37
Energy efficiency							
DEI/GEI	0.62^b^	0.68^a^	0.64^ab^	0.64^ab^	0.013	0.64	0.04

SEM, standard error of the mean; CSC, cassava chip; WBTS, winged bean tuber steam; L, linear; Q, quadratic; GEI, gross energy intake; BW, body weight; DEI, digestible energy intake.

a,b Means within a row with different superscripts differ significantly (p<0.05).
